# Cefepime Neurotoxicity in Acute Kidney Injury: The Importance of Renal Dosing

**DOI:** 10.1155/crin/2274647

**Published:** 2025-04-11

**Authors:** Andrew Lurie, Daniela Carralero Somoza, Graciela Luna, Kaitlyn Cariaga, Umair Syed Ahmed

**Affiliations:** ^1^Department of Internal Medicine, Lakeland Regional Health, Lakeland, Florida 33801, USA; ^2^Department of Nephrology, Lakeland Regional Health, Lakeland, Florida 33801, USA

## Abstract

Cefepime is widely used in sepsis owing to its broad spectrum of activity with coverage against *Pseudomonas*. Though it is usually well-tolerated, in the presence of certain risk factors, renal impairment, high doses, and critical illness, it is associated with the development of neurotoxicity. Those most susceptible are at high risk for developing complications of their underlying illness and the clinical manifestations of cefepime-related neurotoxicity are nonspecific, with reduced level of consciousness, confusion, myoclonus, and seizures, thus a high index of suspicion is required to make a diagnosis. The management includes drug discontinuation, seizure prophylaxis in the right clinical setting, and hemodialysis in severe cases. This case reinforces the need to renally adjust medications in the setting of acute kidney injury to avoid uncommon complications while using common medications.

## 1. Introduction

Cefepime is a fourth-generation cephalosporin, a broad-spectrum antibiotic with activity against both Gram-positive and Gram-negative bacteria including *Pseudomonas aeruginosa*. It is commonly employed alongside agents with activity against methicillin-resistant *Staphylococcus aureus* in the management of sepsis secondary to pneumonia, urinary tract infections, skin and soft tissue infections, intra-abdominal infections, and febrile neutropenia. Though it is generally well-tolerated, all drugs have adverse effects. Similar to other beta-lactams, cefepime has been observed to induce neurotoxicity in some patients.

Here, we report the case of cefepime-related neurotoxicity in a postnephrectomy patient secondary to impaired renal function.

## 2. Case Presentation

The patient was a 68-year-old female with a past medical history of Type 2 diabetes mellitus, hyperlipidemia, emphysematous pyelonephritis status postunilateral nephrectomy, obstructive uropathy, and history of recent acute kidney injury superimposed on chronic kidney disease who presented with generalized weakness, vomiting, and diarrhea. Physical examination was unremarkable, including notably an unremarkable mental status examination. Laboratory findings (Tables 1, 2, and 3) were significant for potassium 2.9 mmol/L, creatinine 716 μmol/L, and blood urea nitrogen (BUN) 28.5 mmol/L. Urinalysis showed microscopic hematuria, pyuria, and proteinuria. Baseline creatinine had been 88 μmol/L until a significant acute kidney injury 4 months prior to admission after which plasma creatinine stabilized at 325 μmol/L.

On hospital admission, the patient was started on cefepime empirically for urinary tract infection, though over the next 3 days, the patient developed worsening encephalopathy ultimately requiring intubation for airway protection. Computed tomography (CT) and magnetic resonance imaging (MRI) of the brain were negative. Electroencephalography revealed nonconvulsive status epilepticus. Seizures were aborted with lorazepam and continued EEG monitoring revealed intermittent generalized periodic discharges with triphasic morphology and an epileptic focus over the left posterior temporal region prompting initiation of levetiracetam for maintenance. A review of medications revealed cefepime had not been dose-adjusted for renal impairment and the patient had been receiving 1000 mg twice daily. Differentials for altered mental status included uremia versus cefepime-associated neurologic toxicity. Cefepime-associated neurologic toxicity was considered more likely as BUN on admission was significantly higher at 28.57 mmol/L with a normal mental status compared to 17.5 mmol/L on the day dialysis was started with altered mental status and seizure activity. Blood cefepime levels were not ordered as this is a send-out test and would have taken several days to be reported and therefore are of no clinical value to us immediately. The patient received two consecutive days of fours of hemodialysis with blood flow rates of 400 mL/minute and dialysate flow rates of 700 mL/minute, respectively.

Renally adjusted meropenem was substituted for cefepime and hemodialysis was initiated. The patient was successfully extubated and recovered to her baseline after two dialysis sessions over the next 2 days. She remained seizure-free and maintained baseline mentation over the subsequent hospital admission despite BUN levels rising to 19.28 mmol/L, which was above her seizure days BUN level of 17.5 mmol/L.

## 3. Discussion

Cefepime is a fourth-generation cephalosporin with a wide spectrum of activity. It has efficacy covering Gram-positive bacteria including *Streptococcus* (pneumonia, *pyogenes*, and *viridans* group) and *Staphylococcus* (methicillin-sensitive); Gram-negative bacteria including *Klebsiella, Haemophilus, Escherichia,* and *Pseudomonas*; and limited anaerobic bacteria activity against *Bacteroides fragilis*. Combination therapy of cefepime plus an antimicrobial covering methicillin-resistant *Staphylococcus aureus* is commonly used in the initial management of sepsis. Cefepime is generally well-tolerated and discontinued voluntarily in place of more narrow-spectrum antibiotics. Nevertheless, the risk of adverse effects remains and deserves consideration prior to initiating therapy, namely, cefepime-induced neurotoxicity.

Cefepime-induced neurotoxicity was first reported in the literature in 2000 [1]. It primarily presents with new or worsened encephalopathy and may be accompanied by other neurologic findings such as myoclonus [2] starting a median of 4 days after initiation of cefepime [3]. If not recognized and the predisposing factors are not ameliorated, mentation will deteriorate further and patients will progress to convulsive or nonconvulsive status epilepticus [4]. Unfortunately, despite increased traction in the literature, there is still wide variability in some reported data. For example, the incidence has a wide-reported range with various studies having demonstrated between 0.2% and 15% of patients developing cefepime-induced neurotoxicity [5–8]. In light of the known deficiencies in our knowledge, increasingly stringent studies of its pharmacologic properties followed to further understand the pathophysiology and guide management to prevent this adverse effect.

Cefepime has a large volume of distribution, penetrates the lung parenchyma, abdominal space, and central nervous system well, and is bound 20% by plasma proteins. It is cleared mainly via glomerular filtration and 85% is excreted unchanged in the urine. Importantly, half-life increases and clearance decreases proportionally with renal dysfunction [9], and it is cleared rapidly during hemodialysis [10]. Despite these significant advances in our knowledge, the pathogenesis underlying neurotoxicity has not yet been fully elucidated. The leading hypothesis is that increased plasma concentration coupled with blood–brain barrier dysfunction in the setting of inflammation allows the infiltration of the central nervous system and inhibition of GABA_A_ receptors causing increased neuron excitability [4, 11, 12].

Certain patient characteristics in those who develop neurotoxicity have been identified pervasively throughout the literature and follow the pharmacology well: renal impairment, critical illness, and high dosing or prolonged treatment [3, 6]. Notably renal impairment is of paramount importance and is often coupled with high-dose therapy; therefore, it may be the most manageable risk factor in light of the mechanism of drug clearance. Given that 85% of cefepime is excreted in the urine unchanged, impaired clearance may quickly increase serum levels. For this reason, renally adjusting medications is of utmost importance in the management of any hospitalized patient with renal impairment. As seen in the patient discussed here, the cefepime dose was not renally adjusted, predisposing the patient to adverse effects secondary to high-dose therapy in the setting of renal impairment. Moreover, critical illness poses a diagnostic dilemma: critically ill patients often have multiple potential explanations for altered mental status. Therefore, a high index of suspicion is required to consider cefepime as the culprit. In our patient, cefepime neurotoxicity was considered to be a more likely cause of her seizures and altered mental status than uremia as her mental status prior to this episode and later was normal despite much higher BUN levels.

The prognosis of a patient with cefepime-related neurotoxicity may be dependent on an early diagnosis. Given the nonspecific nature of this clinical presentation, diagnosis may be missed or delayed, resulting in possible worse outcomes.

Discontinuation of cefepime is the most common intervention needed in patients who manifest neurotoxic side effects. Given the propensity for nonconvulsive epileptiform activity, electroencephalogram monitoring may be considered as well. For those with seizure activity, the cornerstones of therapy are benzodiazepines for abortive therapy and antiepileptic drugs for maintenance. Extracorporeal techniques may sometimes be needed for rapid drug removal. Removal of drugs with dialysis is determined by a complex interaction of many factors. Factors facilitating rapid drug removal include low molecular weight, low protein binding, and low volume of distribution.

Dialysis is dependent upon the use of a dialytic membrane: either a synthetic membrane with fixed pore size, such as in hemodialysis, or a naturally occurring peritoneal membrane, such as in peritoneal dialysis. The movement of drugs or other solutes is largely determined by the size of these molecules in relation to the pore size of the membrane. Smaller molecular-weight substances will pass through the membrane more easily than larger molecular-weight substances. Cefepime has a low molecular weight of around 500 Da, therefore making it easy for it to be removed across dialysis membranes.

Another factor which determines drug removal with dialysis is the concentration gradient of the unbound (free) drug across the dialysis membrane. Drugs with a high degree of protein binding will have a small plasma concentration of unbound drug available for dialysis. Cefepime has low protein binding of 16%–19% and can therefore be effectively cleared with dialysis [13].

Continuous ambulatory peritoneal dialysis (CAPD) is less efficient at clearance of cefepime compared to hemodialysis, with clearances reported to be only 9% of that reported with hemodialysis [9]. Drug clearance of 40%–80% has been reported with hemodialysis using high flux dialyzers [10, 12, 13].

Cefepime-related neurotoxicity was suspected in our patient given the lack of other obvious causes and the temporal relationship of symptoms to initiation of high-dose IV cefepime. Uremic encephalopathy was less likely as BUN levels had remained stable throughout this hospital stay prior to the initiation of hemodialysis.

Given the risk of cefepime-related neurotoxicity, renal dose adjustments have been recommended by the Food and Drug Administration [14].

## 4. Conclusion

Cefepime-related neurotoxicity is an underreported entity. It occurs mostly with incorrect dosing especially in the setting of renal dysfunction. It however may also be seen in patients with appropriate dosing. It is therefore important to consider drug toxicity as a differential in patients with neurological signs and symptoms.

A high index of suspicion is required for clinicians especially in patients with predisposing risk factors, as the prognosis could be dependent on an early recognition of this condition with subsequent discontinuation of the drug. Extracorporeal drug removal with dialysis may be needed to facilitate drug removal and improvement in neurological status.

## Figures and Tables

**Table 1:** Serum laboratory values on admission.

Laboratory	Value (reference range)	Unit
White blood cell count	9.88 (3.40–9.60)∗10^3^	Cells per microliter
Hemoglobin	11.0 (11.6–15)	Grams per liter
Hematocrit	34.5 (35.5–44.9)	Percent
Platelets	273 (157–371)∗10^3^	Cells per microliter
Sodium	136 (136–145)	mmol per liter
Potassium	2.9 (3.5–5.1)	mmol per liter
Chloride	97 (98–107)	Mmol per liter
CO_2_	18 (22–29)	mmol per liter
Blood urea nitrogen	31.07 (2.9–7.1)	mmol/L
Creatinine	716 (62–115)	μmol/L
Aspartate aminotransferase	23 (10–35)	Units per liter
Alanine aminotransferase	46 (10–35)	Units per liter
Glycated hemoglobin	6.6 (4–6)	Percent

**Table 2:** Urine dipstick on admission.

Laboratory	Value (reference range)	Unit
Glucose	Negative (negative)	None
Ketones	Negative (negative)	None
Specific gravity	1.025 (1.007–1.025)	None
pH	6.0 (4.5–8)	None
Blood	4+ (negative)	None
Protein	3+ (negative)	None
Nitrite	Negative (negative)	None
Leukocyte esterase	3+ (negative)	None

**Table 3:** Urine microscopic analysis on admission.

Laboratory	Value (reference range)	Unit
Red blood cells	Many (none)	None
White blood cells	Too numerous to count (none)	None
Bacteria	Few (none)	None
Epithelial cells	None (none)	None

## Data Availability

The data that support the findings of this study are available from the corresponding author upon reasonable request.

## References

[1] Jallon P., Fankhauser L., Du Pasquier R. (2000). Severe but Reversible Encephalopathy Associated With Cefepime. *Neurophysiologie Clinique/Clinical Neurophysiology*.

[2] Ajibola O., Aremu T. O., Dada S. O. (2023). The Trend of Cefepime-Induced Neurotoxicity: A Systematic Review. *Cureus*.

[3] Saini T., Gaines M. N., Sohal A., Li L. (2021). Cefepime-Induced Neurotoxicity. *Cureus*.

[4] Khorasani-Zadeh A., Greca I., Gada K. (2020). Cefepime-Induced Seizures: The Overlooked Outpatient Adverse Reaction. *Cureus*.

[5] Appa A. A., Jain R., Rakita R. M., Hakimian S., Pottinger P. S. (2017). Characterizing Cefepime Neurotoxicity: A Systematic Review. *Open Forum Infectious Diseases*.

[6] Garces E. O., Andrade de Anzambuja M. F., da Silva D., Bragatti J. A., Jacoby T., Saldanha Thomé F. (2008). Renal Failure Is a Risk Factor for Cefepime-Induced Encephalopathy. *Journal of Nephrology*.

[7] Jeon J. Y., Cho Y. W., Moon H. J. (2020). Cefepime-Induced Encephalopathy in a Tertiary Medical Center in Korea. *Journal of Clinical Neurology*.

[8] Fugate J. E., Kalimullah E. A., Hocker S. E., Clark S. L., Wijdicks E. F., Rabinstein A. A. (2013). Cefepime Neurotoxicity in the Intensive Care Unit: A Cause of Severe, Underappreciated Encephalopathy. *Critical Care*.

[9] Barbhaiya R. H., Knupp C. A., Forgue S. T., Matzke G. R., Guay D. R., Pittman K. A. (1990). Pharmacokinetics of Cefepime in Subjects with Renal Insufficiency. *Clinical Pharmacology & Therapeutics*.

[10] Schmaldienst S., Traunmüller F., Burgmann H. (2000). Multiple-Dose Pharmacokinetics of Cefepime in Long-Term Hemodialysis With High-Flux Membranes. *European Journal of Clinical Pharmacology*.

[11] Durand-Maugard C., Lemaire-Hurtel A. S., Gras-Champel V. (2012). Blood and CSF Monitoring of Cefepime-Induced Neurotoxicity: Nine Case Reports. *Journal of Antimicrobial Chemotherapy*.

[12] Mani L. Y., Kissling S., Viceic D., Vogt B., Burnier M. (2015). Intermittent Hemodialysis Treatment in Cefepime‐induced Neurotoxicity: Case Report, Pharmacokinetic Modeling, and Review of the Literature. *Hemodialysis International*.

[13] Descombes E., Martins F., Hemett O. M., Erard V., Chuard C. (2016). Three-times-weekly, Post-dialysis Cefepime Therapy in Patients on Maintenance Hemodialysis: a Retrospective Study. *BMC Pharmacology Toxicology*.

[14] Hospira Inc (2024). Maxipime [package Insert]. *U.S. Food and Drug Administration website*.

